# Guiding the starting dose of the once-daily formulation of tacrolimus in “*de novo”* adult renal transplant patients: a population approach

**DOI:** 10.3389/fphar.2024.1456565

**Published:** 2024-09-19

**Authors:** Beatriz Fernández-Alarcón, Oscar Nolberger, Anna Vidal-Alabró, Raul Rigo-Bonnin, Josep M. Grinyó, Edoardo Melilli, Nuria Montero, Anna Manonelles, Ana Coloma, Alex Favà, Sergi Codina, Josep M. Cruzado, Helena Colom, Nuria Lloberas

**Affiliations:** ^1^ Nephrology Department, Hospital Universitari de Bellvitge-IDIBELL, Barcelona, Spain; ^2^ Biopharmaceutics and Pharmacokinetics Unit, Department of Pharmacy and Pharmaceutical Technology and Physical Chemistry, School of Pharmacy and Food Sciences, University of Barcelona, Barcelona, Spain; ^3^ Biochemistry Department, Hospital Universitari de Bellvitge-IDIBELL, Barcelona, Spain; ^4^ Medicine Unit, Department of Clinical Sciences, University of Barcelona, Barcelona, Spain

**Keywords:** age, CYP3A4, CYP3A5, *de novo*-kidney transplant patients, ER-Tac, hematocrit, population pharmacokinetics

## Abstract

**Aims:**

The once-daily extended-release tacrolimus formulation (ER-Tac) has demonstrated similar efficacy and safety to the twice-daily immediate-release formulation (IR-Tac), but few population-based pharmacokinetic models have been developed in *de novo* kidney transplant patients to optimize doses. Therefore, this study aimed i) at developing a population pharmacokinetic model for ER-Tac in *de novo* adult kidney transplant patients ii) and identifying genetic factors and time-varying covariates predictive of pharmacokinetic variability to guide tacrolimus dosage during the early post-transplant period.

**Methods:**

A total of 1,067 blood tacrolimus concentrations from 138 kidney transplant patients were analyzed. A total of 29 out of 138 patients were intensively sampled for 24 h on the day 5 post-transplantation; meanwhile, for the remaining patients, concentrations were collected on days 5, 10, and 15 after transplantation. Tacrolimus daily doses and genetic and demographic characteristics were retrieved from the medical files. Biochemistry time-varying covariates were obtained on different days over the pharmacokinetic (PK) study. A simultaneous PK analysis of all concentrations was carried out using the non-linear mixed-effects approach with NONMEM 7.5.

**Results:**

A two-compartment model with linear elimination and delayed absorption best described the tacrolimus pharmacokinetics. Between-patient variability was associated with oral blood clearance (CL/F) and the central compartment distribution volume (Vc/F). Tacrolimus concentrations standardized to a hematocrit value of 45% significantly improved the model (*p* < 0.001). This method outperformed the standard covariate modeling of the hematocrit–blood clearance relationship. The effect of the CYP3A5 genotype was statistically (*p* < 0.001) and clinically significant on CL/F. The CL/F of patients who were CYP3A5*1 carriers was 51% higher than that of CYP3A5*1 non-carriers. Age also influenced CL/F variability (*p* < 0.001). Specifically, CL/F declined by 0.0562 units per each increased year from the value estimated in patients who were 60 years and younger.

**Conclusion:**

The 36% between-patient variability in CL/F was explained by CYP3A5 genotype, age, and hematocrit. Hematocrit standardization to 45% explained the variability of tacrolimus whole-blood concentrations, and this was of utmost importance in order to better interpret whole-blood tacrolimus concentrations during therapeutic drug monitoring. The dose requirements of CYP3A5*/1 carriers in patients aged 60 years or younger would be highest, while CYP3A5*/1 non-carriers older than 60 years would require the lowest doses.

## 1 Introduction

Tacrolimus (Tac) is a calcineurin inhibitor that is currently used as an immunosuppressant in renal transplantation to prevent the organ from being rejected (Venkataramanan et al., 1995; Staatz and Tett, 2004). Its narrow therapeutic index and high inter- and intra-individual variability have led to many publications addressing the importance of population pharmacokinetic models as support tools for dose adjustment (Zhao et al., 2016; Andrews et al., 2018; Campagne et al., 2019; Kirubakaran et al., 2022). The once-daily extended-release tacrolimus (ER-Tac) (Bowman and Brennan, 2008) was developed to improve drug compliance, gastrointestinal tolerability, and reduce pill burden. The conversion from twice-daily immediate-release tacrolimus (IR-Tac) to once-daily ER-Tac proved to be effective and safe in the short (Krämer et al., 2010; Caillard et al., 2016) and middle terms (Spagnoletti et al., 2014). Several published studies have characterized the Tac exposure for ER-Tac (Staatz and Tett, 2015), compared it with that provided by IR-Tac (Wlodarczyk et al., 2009; Krämer et al., 2010; Tremblay and Alloway, 2017), and identified several factors predictive of inter-individual pharmacokinetic variability (Campagne et al., 2019). In this regard, in contrast to IR-Tac, few population-based PK studies have been described in the literature for ER-Tac. A recent search on PubMed yielded over 60 publications related to population PK models of the IR-Tac formulation, with many of them reported in current reviews (Kirubakaran et al., 2020; 2022). However, very few studies have addressed the ER-Tac formulation (Lu et al., 2019; Benkali et al., 2010; Saint-Marcoux et al., 2010; Woillard et al., 2011; Zhao et al., 2013), and even fewer have focused on *de novo* kidney transplant patients. Simultaneous pharmacokinetic modeling of tacrolimus concentration–time data in both the early and subsequent post-transplant phases should allow for the characterization of time-varying pharmacokinetics throughout the complete post-transplant period. Nevertheless, attempts to achieve this have not fully described all changes, especially in CL/F, over the whole post-transplant period (Bergmann et al., 2014; Zhang et al., 2017). Hematocrit is known to be one of the factors that contribute to changes in Tac CL/F and in Tac blood concentrations along all the post-transplant time (Staatz and Tett, 2004; Campagne et al., 2019). The influence of hematocrit on Tac blood concentrations is due to its high binding ability to erythrocytes and to the Tac restrictive clearance. Kidney transplant patients have usually low hematocrit fractions in the immediate post-operative period, which increases up to the stable phase. In most centers, therapeutic drug monitoring is based on trough Tac blood concentrations (C_trough_), so it is important to take into account the influence of hematocrit in improving tacrolimus dose calculation in the early post-transplant phase. Some studies have included post-transplant time as an additional covariate, alongside hematocrit, corticosteroids, and other factors. However, this approach comes with its own set of challenges: i) difficulties arise in calculations when drug disposition processes occur on a shorter timescale than the 1-year post-transplant period, and ii) frequently, the proportion of concentration–time data on the late post-transplant period is larger compared to the earliest phase. These issues lead to considering the development of a model only including *de novo* kidney transplant patients. On the other hand, the impact of genetic polymorphisms of CYP3A5 and CYP3A4 compared to other individual patient factors such as body size, composition, or age remains to be evaluated in *de novo* kidney transplant patients treated with ER-Tac. Dosing based on these factors can contribute greatly to reducing the risk of rejection and adverse effects (Brunet et al., 2019).

Therefore, the aims of the current study are i) to develop a population pharmacokinetic model of tacrolimus in *de novo* adult kidney transplant patients receiving the ER-Tac once-daily formulation and ii) identify the genetic factors and time-varying covariates that could predict pharmacokinetic variability during the early post-transplant period in order to guide tacrolimus dosage adjustments.

## 2 Methods

### 2.1 Study design, patients, and treatment

A total of 138 adult renal transplant patients who received both—living and deceased—donor grafts at Hospital Universitari de Bellvitge (Barcelona, Spain) in 2012–2022 were enrolled in this study. The patients received an extended-release, once-daily administered, formulation of Tac, Advagraf^®^ (Astellas Pharma, Europe Ltd., Staines, United Kingdom), as *de novo* immunosuppressant therapy for at least 3 months post-transplantation with a dosing regimen of 0.1 mg/kg/day initiated 24 h after transplantation. Physicians monitored and adjusted the Tac doses to achieve the predefined target whole-blood concentration of 5–10 ng/mL. The study protocol for this retrospective observational study was approved by the Comité de Ética de la Investigación con Medicamentos del Hospital Universitari de Bellvitge (Ref. EOM009/22). The study was carried out following the WMA Declaration of Helsinki, and all patients provided informed written consent prior to inclusion.

As part of the triple immunosuppressant therapy, patients also received mycophenolic acid (CellCept^®^, Myfortic^®^) with a dosing regimen ranging from 500 to 2,000 mg per day and corticosteroids. Initially, the corticosteroid dose was set between 5 and 10 mg per day and then was subsequently tapered down over several months to either 5 mg per day or discontinued entirely.

Patients were excluded if they were receiving medications that induce or inhibit CYP3A enzymes. Additionally, patients with hepatic diseases, hypersensitivity to Tac, severe diarrhea, vomiting, active peptic ulcers, or other gastrointestinal diseases that could affect Tac absorption were excluded. Patients with hepatitis B or C, HIV, or neoplasms and pregnant and lactating women were also excluded. Furthermore, patients were excluded if DNA extraction could not be performed due to lack of availability or sample quality.

### 2.2 Blood sampling and data recording

The patient cohort consisted of 138 patients, and a total of 1,067 blood Tac concentrations were analyzed. Among these, 29 out of 138 patients underwent extensive analysis including a full pharmacokinetic profile over 24 h collected on the day 5 post-transplantation while still hospitalized. On this day, whole-blood samples were drawn before administration (trough value) and at 0.5, 1, 2, 4, 8, 12, 20, and 24 h after administration (n = 261 concentration–time values). Additionally, trough blood concentrations were determined on days 10 and 15 after transplantation (n = 233 concentration–time values). For the remaining patients, trough blood concentrations were collected on days 5, 10, and 15 after transplantation (n = 573 concentration–time values). Tacrolimus daily doses and demographic characteristics of the patients were retrieved from the medical files at the initiation of treatment. Biochemical characteristics including serum creatinine (µmol.L^-1^), glomerular filtration rate (calculated with Chronic Kidney Disease Epidemiology Collaboration, CKD-EPI formula), albumin, alanine aminotransferase, gamma-glutamyl transferase, proteinuria, and hematocrit (%) were continuously monitored at each occasion along with concentrations.

### 2.3 Tacrolimus measurement

Tacrolimus was measured using a LC–MS/MS method that was previously developed and validated^30^. Chromatographic determination was performed using the Acquity (®) UPLC (®) with a C18 BEH™ reversed phase column (2.1 × 50 mm id, 1.7 μm). The limit of quantitation was set at 1.0 ng/mL.

### 2.4 Genotyping

For genotyping, the patients’ whole blood was sampled and then used for DNA extraction using Maxwell RSC^®^ (Promega Corporation, Sydney, Australia) and stored at −80°C. To perform genotyping for the identification of the SNPs CYP3A5*3 A>G (rs776746), CYP3A4*22 C>T (rs35599367), and ABCB1 3435C>T (1045642), specific TaqMan SNP Genotyping Assays (Applied Biosystems, Foster City, CA, United States) were used. Samples were analyzed by real-time PCR using the 7900HT Fast Real-time PCR System, Applied Biosystems (Thermo Fisher Scientific, Waltham, MA, United States). All analyses were conducted in accordance with the manufacturer’s instructions. After genotyping, alleles were classified so that they could be used for the exploratory analysis and model building. Specifically, CYP3A4 genotypes were grouped as CYP3A4 *1/*1 and CYP3A4*22 carriers (*1/*22 and *22/*22). Concerning CYP3A5, CYP3A5*1 carrier (*1/*1 and *1/*3) and *CYP3A5* non-carrier (*3/*3) groups were considered*.* Regarding the functional metabolic rate associated with the CYP3A4 and CYP3A5 variants, patients were classified into three groups: poor metabolizers (PM; CYP3A4*22 carriers + CYP3A5*3/*3), intermediate metabolizers (IM; CYP3A4 *1/*1 + CYP3A5 non-carriers or CYP3A4*22 carriers + CYP3A5*1 carriers), or extensive metabolizers (EM; CYP3A4 *1/*1 + CYP3A5*1 carriers) (Lloberas et al., 2017; Andreu et al., 2017).

### 2.5 Statistical analysis

Demographic and biochemistry continuous variables were presented in tables with median, minimum, and maximum values. Categorical variables such as clinical and genetic characteristics were given as the number of observations and percentages. Trough concentrations (C_trough_) were reported as geometric means and 95% confidence intervals. Global mean biochemistry time-varying variables were calculated along with mean values at each occasion on days 5, 10, and 15 post-transplantation.

C_trough_ were the observed values just before each given dose. The areas under the curve from zero to 24 h (AUC_24_) were calculated using the linear-log trapezoidal rule of the non-compartmental analysis with the Phoenix WinNonlin version 840.6172. Normalised by dose AUC_24_ and C_trough_ values (AUC_24_/Dose and C_trough_/Dose) were also calculated and presented as geometric means (95% confidence interval). AUC_24_/Dose and C_trough_/Dose as the time-varying covariates were compared statistically with a three-way analysis of variance, considering the occasion (days 5, 10, or 15) and genetic variant as fixed factors and the patient as a random factor nested within the genetic variant. Log-transformed values of AUC_24_/D and C_trough_/D were used according to normal practice (Davit et al., 2013). R package (version 4.3.2) was used in all the statistical comparisons, and statistical significance was set at *p* < 0.05.

### 2.6 Population pharmacokinetic analysis

The population pharmacokinetic (PopPK) model was developed using the nonlinear mixed-effects modeling approach implemented in NONMEM^®^ version 7.5 (ICON Development Solutions, Hanover, MD, United States). Perl-Speaks-NONMEM (PsN) version 5.5., R package version 4.3.2, Pirana Modeling Workbench version 3.0 (Certara L.P. (Pharsight), St. Louis, MO), and Xpose 4.7.2 were used for data management, exploratory data analysis, plotting graphical outputs, and model evaluation, respectively. The first-order conditional estimation (FOCEI) method with an interaction was used throughout the modeling process.

#### 2.6.1 Base model development

One- and two-compartment open models with linear elimination and first-order delayed absorption were tested to describe the Tac blood concentration–time data. Classical lag-time models and transit compartment models, using either Erlang or gamma distribution kinetic profiles, were tested to describe the delayed absorption process (Savic et al., 2007). The models were parameterized in terms of apparent blood elimination clearance (CL/F), apparent central and peripheral compartment distribution volumes (Vc/F and Vp/F), and apparent inter-compartmental clearance (CL_D_/F), absorption rate constant (Ka), and lag time (ALAG1) for classical lag time models. For transit compartment models, mean transit time (MTT) and the number of compartments (NN) were applied. The bioavailability (F) was set at 1 due to the lack of intravenous data. Between-patient variability (BPV) was tested for each pharmacokinetic parameter using an exponential error model that assumes log-normal distributions. Inter-occasion variability (IOV) was applied and evaluated for elimination clearance (Karlsson and Sheiner, 1993; Abrantes et al., 2019). Additive, proportional, and combined error models were assessed to describe the residual error (RE) variability.

To identify statistical superiority between the nested models, the likelihood ratio test was used, which was based on the reduction of the minimum objective function value (MOFV). A significance level of *p* < 0.005 was applied, corresponding to ΔMOFV = −7.879 for 1 degree of freedom. For the non-hierarchical models, the most parsimonious model with the lowest MOFV according to the Akaike information criterion (AIC) was chosen (Yamaoka et al., 1978). The decrease in MOFV, parameter precision given by relative standard error expressed as percentage (RSE%), reductions in BPV, η-, and ε-shrinkage (Savic and Karlsson, 2009) values, model completion status, and condition number indicative of correlations among parameters were examined. Additionally, the visual inspection of goodness of fit plots was considered to evaluate the model selection and descriptive capability.

#### 2.6.2 Covariate analysis

The influence of the most physiologically or clinically meaningful covariates on the pharmacokinetic parameters was assessed. For that purpose, Bayesian estimates of the pharmacokinetic parameters were plotted against several covariates at an initial stage. Estimated demographic, biochemical, and genetic covariates included age, gender, fat-free mass, bodyweight, body mass index, hematocrit, and serum creatinine. Additionally, genotypes for CYP3A5, CYP3A4, and ABCB1 were analyzed. Specifically, CYP3A5 variants (CYP3A5*1 carriers vs. non-carriers), CYP3A4 variants (CYP3A4*22 carriers vs. non-carriers), and *ABCB1* variants (*C/*C, *C/*T, and *T/*T) were tested. The three metabolizer phenotypes based on CYP3A4 and CYP3A5 genotypes (EM, IM, and PM), which were previously described, were also tested in the model (Andreu et al., 2017).

Covariates were initially explored univariately and then using the established forward–backward stepwise approach. A significance level of *p* < 0.05 corresponding to ΔMOFV = −3.841 was considered during the forward stepwise addition, while a significance level of *p* < 0.001 corresponding to a ΔMOFV increase of 10.83 (when a significant covariate was removed) was used during the backward elimination for model refinement. Only covariates providing a 10% reduction in parameter BPV were considered clinically significant and remained as part of the model. In addition to the statistical criteria, the physiological theory-based criteria for entering a covariate were considered. Continuous covariates such as age, fat-free mass, bodyweight, creatinine, and renal function were systematically tested one at a time and introduced as a power function (Equation 1) in the model.

$$\text{TVP}j=\theta_{1}\;{\left(\frac{\text{COV}}{{\text{COV}}_{\text{median}}}\right)}^{\theta \text{COV}},$$ (1)

where θ_1_ is the typical value of the *j*th pharmacokinetic parameter (TVP*j*) for a patient whose covariate value (COV) is equal to the population median (COV_median_) and θCOV is the change in lnTVP*j* per unit change in ln (COV/COV_median_). As before (Størset et al., 2014a; Størset et al., 2014b), fat-free mass was calculated according to equations previously reported (Sinha et al., 2020) and evaluated in all the disposition parameters considering the allometric scaling by fixing the exponents at 0.75 and 1 for the flow parameters (clearances) and distribution volumes, respectively (Holford and Anderson, 2017). Categorical covariates (CYP3A4, CYP3A5, CYP3A cluster, ABCB1, and gender) were systematically evaluated in the model separately, according to Equation 2.

$$\text{TVP}j=\theta_{1}\text{ for }Z=0,$$

$$\text{TVP}j={\theta_{1}\;\cdot \theta }_{2}\text{ for }Z=1,$$

$$\text{TVP}j={\theta_{1}\;\cdot \theta }_{3}\text{ for }Z=2,$$ (2)

where θ_1_ is the typical value of the *j*th pharmacokinetic parameter (TVP*j*) for a patient categorized to group (Z) equal to 0. θ_2_ and $\theta_{3}$ are the added effects of patients categorized to groups Z equal to 1 or 2 with respect to group Z equal to 0.

As before (Størset et al., 2014a; Størset et al., 2014b; Schijvens et al., 2019), tacrolimus whole-blood concentrations were standardized to a hematocrit of 45% to estimate tacrolimus clearances and distribution volumes in terms of whole-blood concentration standardized to hematocrit 45%. The non-linear binding of tacrolimus distributed into erythrocytes was evaluated, as reported before (Størset et al., 2014a; Størset et al., 2014b).

#### 2.6.3 Model evaluation

Goodness-of-fit plots were analyzed throughout the modeling process. The predictive capability was evaluated using prediction-corrected visual predictive checks (pcVPC) based on 1,000 simulations (Bergstrand et al., 2011). The median and 2.5th and 97.5th percentiles of the simulated data and their respective 95% prediction intervals were calculated and visually compared with the same percentiles obtained from the original raw data. A non-parametric resampling bootstrap procedure with replacement from 500 resampling of the original dataset was applied to further calculate the 95% confidence intervals of all the estimated parameters and the stability of the model.

#### 2.6.4 Monte Carlo simulations of actual dosing regimens

The final model with estimated fixed and random-effects parameters was applied to stochastically simulate 1,000 time–concentration tacrolimus exposure profiles for different once-daily doses and for each level of each covariate identified as statistically and clinically significant in the model. All the suitable combinations of the different levels of the significant covariates were considered. The ranges of once-daily tacrolimus doses were selected on the basis of a 70-kg bodyweight. Increases in steps of 0.5 mg were considered in the simulated doses within each established range. From 1,000 simulated datasets for each dosage regimen/covariate level, AUC_24_ values and trough concentrations were calculated using the non-compartmental approach with Phoenix WinNonlin software version 64.84. These metrics were presented as boxplots. All statistical analyses were performed using R software (version 4.3.2.).

## 3 Results

### 3.1 Patient characteristics and datasets

A total of 1,067 tacrolimus whole-blood concentration–time values were used for the development of the model: 634 sets of values were collected from the intensively sampled patients (n = 29), and 1,408 were C_trough_ values obtained from all patients included in the study (n = 138). All whole-blood concentrations were over the limit of quantification. Demographic, biochemistry, clinical, and genetic characteristics of the patient population are summarized in Table 1. All patients underwent genotyping for CYP3A4*22, CYP3A5*3, and *ABCB1 3435C>T.* However, no individuals with the CYP3A4*22/*22 genotype were found. Glomerulonephritis was the primary cause of transplantation, and 61% of the transplants was performed with organs from living donors.

**TABLE 1:** Patient demographic and clinical and genetic characteristics at baseline. Median (min–max) values are shown for continuous variables. Categorical variables are given as N (%).

Characteristic	Value
Number of patients (N)	138
Number of doses (N)	2,309
Number of doses per patient (N)	17
Doses (mg) Doses (mg/kg)	6 (0.5–23) 0.091 (0.0068–0.211)
Gender (male/female) (N/N)	90/48
Age (years)	54 (19–82)
Weight (kg)	70.5 (44.0–109.0)
BMI (kg/m^2^)	25.6 (17.2–36.2)
Fat-free mass (kg)	51.9 (30.5–73.9)
Type of transplant	
Alive donor organ	84 (61%)
Cadaver donor organ	54 (39%)
Kidney disease	
Glomerulonephritis	38 (27.5%)
Interstitial nephritis	21 (15.2%)
Polycystic kidney disease	19 (13.7%)
Vascular origin	17 (12.3%)
Diabetes nephropathy	12 (8.7%)
Urologic origin	2 (1.5%)
Unknown origin	29 (21.1%)
CMV positive	109 (79%)
Donors	
Gender (M/F)	64/74
Age	55 (27–83)
CMV positive	105 (76%)
CYP3A4 genotype	
*1/*1	126 (91.4%)
*1/*22	12 (8.6%)
*22/*22	0 (0%)
CYP3A5 genotype	
*1/*1	3 (2.2%)
*1/*3	17 (12.3%)
*3/*3	118 (85.5%)
CYP3A cluster$	
Poor metabolizer	10 (7.3%)
Intermediate metabolizer	110 (79.7%)
Extensive metabolizer	18 (13.0%)
ABCB1 genotype	
*C/*C	38 (27.5%)
*C/*T	72 (52.2%)
*T/*T	28 (20.3%)
HLA mismatches	
AB-mismatches 0/1/2/3/4 (%)	9.4/5.8/25.4/39.9/19.5
DR-mismatches 0/1/2 (%)	15.9/55.8/28.3
Mycophenolic acid (yes/no)	134/4

Data are presented as the number of cases and percentages (given in parenthesis) for categorical variables and as median (min–max) for continuous variables. M, male; F, female; CMV, cytomegalovirus; min, minimum value; max, maximum value. $CYP3A cluster variable combining the CYP3A5*3 and CYP3A4*22 SNPs, defined as extensive (EMs), intermediate (IMs), and poor metabolizers (PMs); PMs (CYP3A4*22 carriers with the CYP3A5*3/*3 genotype), IMs (CYP3A4*22 non-carriers with the CYP3A5*3/*3 genotype or CYP3A4*22 carriers with the CYP3A5*1/*1 genotype), and EMs (CYP3A4*22 non-carriers and CYP3A5*1 carriers).

**FIGURE 1:**
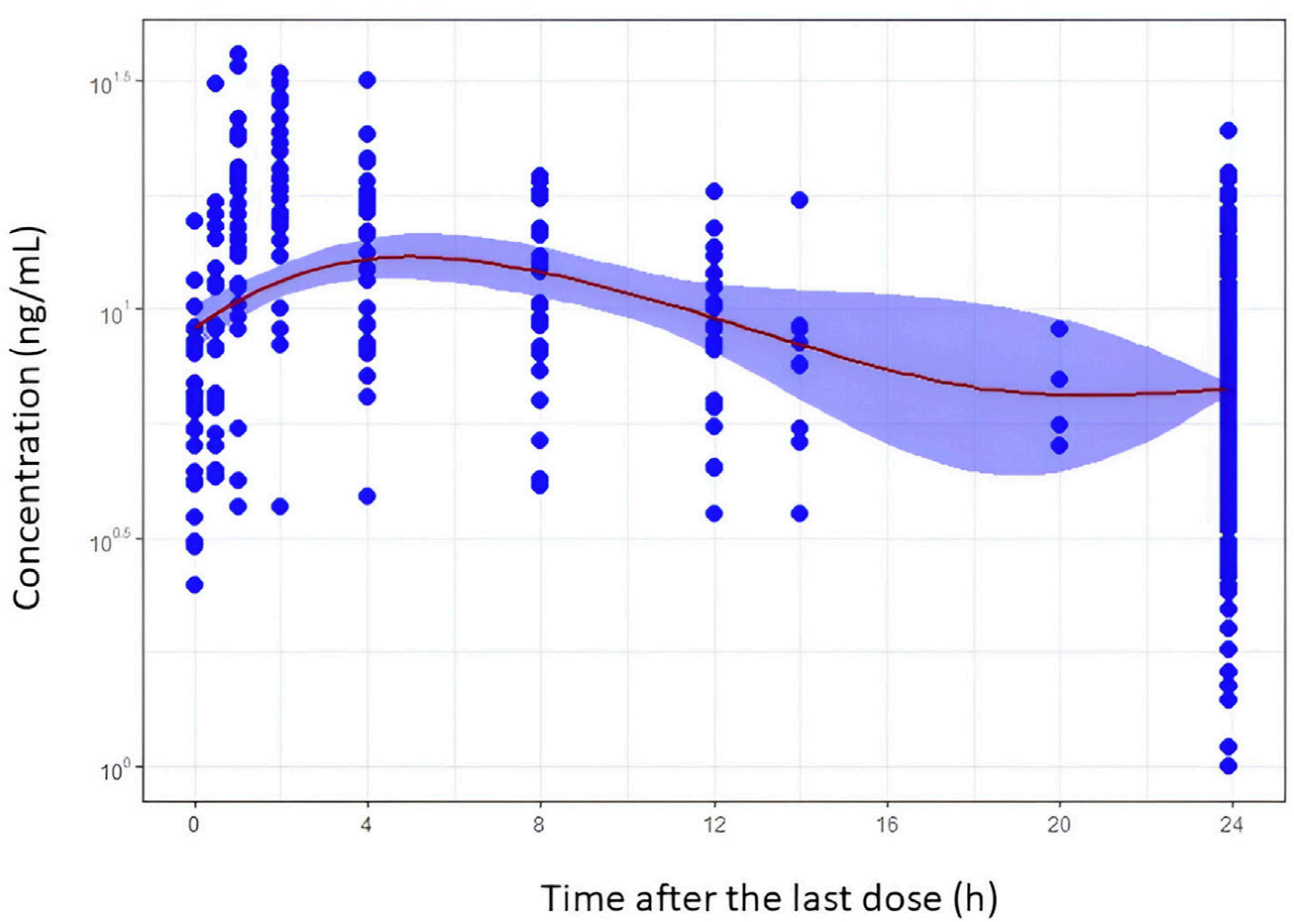
Tacrolimus blood concentration–time profiles after oral administration of the once-daily extended-release formulation in *de novo* kidney transplant patients. Filled blue circles represent the observed concentration. The mean trend of the data is shown by the red solid line that represents a smooth regression using the LOESS non-parametric method. The blue band is the 95% confidence interval of the mean trend.

Table 2 shows the overall medians of the biochemical variables and the medians for each day of study (days 5, 10, and 15). According to these results, the hematocrit, albumin, and renal functions increased significantly with time after transplant. The descriptive statistics of exposure metrics (AUC_24_ and C_trough_) are presented in Table 3. Figure 1 depicts the concentration–time profiles in the whole population. Mean C_trough_ values remained within the range of 5–10 ng/mL. Statistically significant differences were observed in C_trough_/D and AUC_24_/D, between the CYP3A5 genetic groups (i.e., CYP3A5*1 carriers vs. non-carriers) and between extensive and intermediate metabolizers when the influence of the cluster was considered. No statistically significant differences between CYP3A4 and ABCB1 genotype groups were found.

**TABLE 2:** Descriptive statistics of biochemistry variables and trough concentrations (C_trough_) on days 5, 10, and 15 of the study.

Characteristic	Global	5 days	10 days	15 days
Hematocrit (%)	33.2 (20.9–46.6)	29.7 (22.3–46.0)^1,2^	31.3 (20.9–46.6)^1,3^	32.9 (21.7–46.0)^2,3^
Cr (µmol/L)	128 (57–768)	131 (57–768)	127 (57–752)	134 (72–578)
GFR (mL/min)	53 (5–111)	51 (6–111)^7^	53 (5–95)	52 (7–108)^7^
Albumin (g/L)	41 (13–54)	34 (21–54)^4,5^	36 (13–54)^4,6^	41 (25–50)^5,6^
ALT (µkat/L)	0.30 (0.1–4.3)	0.29 (0.1–2.72)	0.38 (0.1–4.3)	0.31 (0.1–4.2)
C_trough_ (ng/mL)	6.65 (6.33–6.98)	5.77 (5.29–6.30)	7.10 (6.50–7.75)	7.35 (6.83–7.91)
C_trough_/Dose (ng/mL/mg)	1.04 (0.99–1.10)	0.90 (0.82–0.98)^8,9^	1.10 (0.99–1.22)^8^	1.18 (1.07–1.31)^,9^

Data are presented as median (min–max) for biochemistry variables. C_trough_ concentrations and normalized by dose C_trough_ are presented as geometric means (95% confidence interval). Cr, serum creatinine; GFR, glomerular filtration rate, estimated according to CKD-EPI formula; ALT, alanine aminotransferase; X^X^, statistical significance between values set at (*p* < 0.05). 1, 2, 3: statistically significant differences between days 5 and 10, (*p* = 0.03), between days 5 and 15 (*p* < 0.001), and between days 10 and 15 (*p* = 0.036), respectively. 4, 5, 6: statistically significant differences between days 5 and 10, between days 5 and 15, and between days 10 and 15, respectively, *p* < 0.001 in all the cases. 7: statistically significant differences between days 5 and 15, *p* = 0.021. 8, 9: statistically significant differences between days 5 and 10 and between days 5 and 15, respectively; *p* < 0.001 in all the cases.

**TABLE 3:** Comparative normalized by dose trough concentrations and AUC_24_ values sorted by the CYP3A5, CYP3A4, ABCB1 genotypes and by the three different cluster phenotypes.

Genotype group	Dose (mg/kg)	C_trough_	N	AUC_24_	N	C_trough_/D	*p*-value*	AUC_24_/D	*p*-value **
		(ng/mL)		(ng.h/mL)					
CYP3A5									
CYP3A5*1/*1, *1/*3	0.103 (0.020–0.282)	4.61 (3.96–5.37)	62	252 (180–353)	26	0.61 (0.52–0.72)	<0.001	22.43 (15.62–32.22)	<0.001
CYP3A5*3/*3	0.091 (0.007–0.211)	7.07 (6.73–7.42)	370	191 (165–223)	3	1.14 (1.08–1.21)		38.21 (32.98–44.28)	
CYP3A4									
CYP3A4*1/*1	0.093 (0.068–0.282)	6.63 (6.29–6.98)	393	207 (178–241)	26	1.04 (0.98–1.10)	0.683	34.88 (29.34–41.46)	0.449
CYP3A4*1/*22	0.097 (0.0208–0.129)	6.84 (5.83–8.03)	39	198 (99–398)	3	1.11 (0.93–1.33)		41.24 (38.70–43.94)	
CLUSTER									
High metabolizer	0.102 (0.020–0.282)	4.49 (3.82–5.29)	56	252 (180–353)	3	0.61 (0.51–0.73)	<0.001^1^ <0.001^2^	22.43 (15.62–32.22)	<0.046^1^ <0.0077^2^
Intermediate metabolizer	0.092 (0.007–0.211)	7.05 (6.70–7.41)	343	190 (161–224)	23	1.12 (1.06–1.19)	0.270^3^	37.70 (31.63–44.93)	0.898^3^
Poor metabolizer	0.097 (0.021–0.120)	7.04 (5.90–8.42)	33	198 (99–398)	3	1.23 (1.03–1.47)		41.24 (38.70–43.94)	
ABCB1									
ABCB1 (*C/*T, *C/*C)	0.090 (0.035–0.136)	6.44 (5.89–7.04)	89	203 (170–242)	6	1.12 (1.00–1.25)	0.486	36.36 (24.98–52.91)	0.4537
ABCB1 (*T/*T)	0.095 (0.007–0.282)	6.70 (6.33–7.10)	343	213 (163–279)	23	1.02 (0.96–1.09)		35.50 (29.66–42.48)	

Values are reported as geometric means (95% CI).

Abbreviations: AUC_24_, area under the whole-blood concentration–time curve; C_trough_, trough whole-blood concentrations, C_trough_/D and AUC_24_/D normalized by dose C_trough_ and AUC_24_ values. N, number of occasions data analyzed is the same for C_trough_ and C_trough_/D. Dose expressed as median (range).

*p-values for C_trough_/D mean values statistical comparisons; 1, 2, 3 statistical significance of comparisons between EM and IM, EM and PM, and IM and PM, respectively.

**p-values for AUC_24_/D mean value statistical comparison.

### 3.2 Population PK analysis

A two-compartment model with delayed first-order absorption and linear elimination from the central compartment best described the tacrolimus blood concentrations. The transit compartment models using gamma distribution provided a good description of the delayed absorption, resulting in a drop of 196 units of the minimum objective function value (MOFV) compared to the classical lag-time model. However, this approach resulted in over-parameterization, leading to ill-conditioning models with collinearity between parameters. This led to difficulties in identifying and calculating all the pharmacokinetic parameters with adequate precision. Before simplifying, the strategies of fixing either the absorption rate constant (Ka) or the number of transit compartments were tested, and it was still insufficient. Consequently, the simplest classical lag-time model was retained. Even with the greater simplicity of the model, the Ka value had to be fixed, and a value of 2 h^-1^ was selected based on a range of physiologically meaningful assayed values. Between-patient variability could be associated with CL/F and Vc/F. The unexplained residual error was modeled as proportional. Standardizing whole-blood concentrations to a hematocrit of 45% reduced the minimum objective function value (MOFV) by 37.7 units (*p* < 0.005). This approach showed itself to be superior to the observed MOFV drop when hematocrit was introduced as a covariate in CL/F. Moreover, BPV associated with CL/F and Vc/F decreased by 15.9% and 10.6%, respectively.

The graphical exploration of inter-individual random effects associated with CL/F from the base model versus CYP3A5 polymorphisms and age suggested a potential relationship (Supplementary Figures S1, S2). Due to the low number of CYP3A5*1/*1 genotype (n = 3) in the dataset, a CYP3A5*1 carrier group (n = 17) was defined for the covariate analysis, as done in the initial statistical analysis. No significant differences between CL/F of intermediate and poor metabolizers (Supplementary Figure S1) were observed according to the initial statistical analysis results (Table 3). Similarly, no differences were seen between the CL/F values of CYP3A4*22 carriers and non-carriers. The inclusion of these covariates in the model led to a statistically significant association between the CYP3A5 genotype and CL/F. This was evidenced by a decrease in the MOFV of 25.84 units (*p* < 0.05) and a 16.5% reduction in the BPV associated with this parameter. Conversely, the inclusion of cluster effects on CL/F did not improve the model. No change was shown in the MOFV compared to the CYP3A5 model. Furthermore, CYP3A4 polymorphism did not explain the differences in CL/F.

The visual inspection of the plots of inter-individual random effects associated with CL/F (estimated from the base model) versus age did not evidence any influence of age on CL/F for patients aged 60 years and younger. However, there was a decrease in CL/F with age, as shown by patients older than 60 years. Therefore, the CL/F–age relationship could not be adequately described by a power model. Instead, it was modeled according to Equation 3; the result shown was a fall in the MOFV by 16.7 units and a reduction in BPV by 16.5%.

$$\text{TVP}j=\theta_{1}-\theta_{2}\cdot AGE\cdot agecat,$$ (3)

where agecat is a categorical index that equals 0 for patients aged 60 years and younger and equals 1 for patients older than 60 years. $\theta_{1}$ is the clearance for patients aged 60 or younger, and $\theta_{2}$ is the reduction in CL/F for each unit of increase in age from $\theta_{1}$ .

The influence of patients’ body size/body composition described by allometric scaling of all the disposition pharmacokinetic parameters based on either bodyweight or fat-free mass was not statistically significant. The inclusion of fat-free mass leads to under-prediction of the peak concentrations. In contrast, allometric scaling of CL/F, Vc/F, and CL_D_/F to 70 kg bodyweight improved the prediction of both peak and trough concentrations. However, the scaling of Vp/F to bodyweight increased the imprecision of between-patient variability associated with Vc/F, and therefore, this relationship was discarded.

The backward elimination procedure confirmed the statistical and clinical influence of genetic polymorphism of CYP3A5 and age in CL/F and of hematocrit in residual error. Therefore, the final model accounted for differences in tacrolimus blood concentrations due to hematocrit variation and the effect of CYP3A5 genetics and age on CL/F.

**FIGURE 2:**
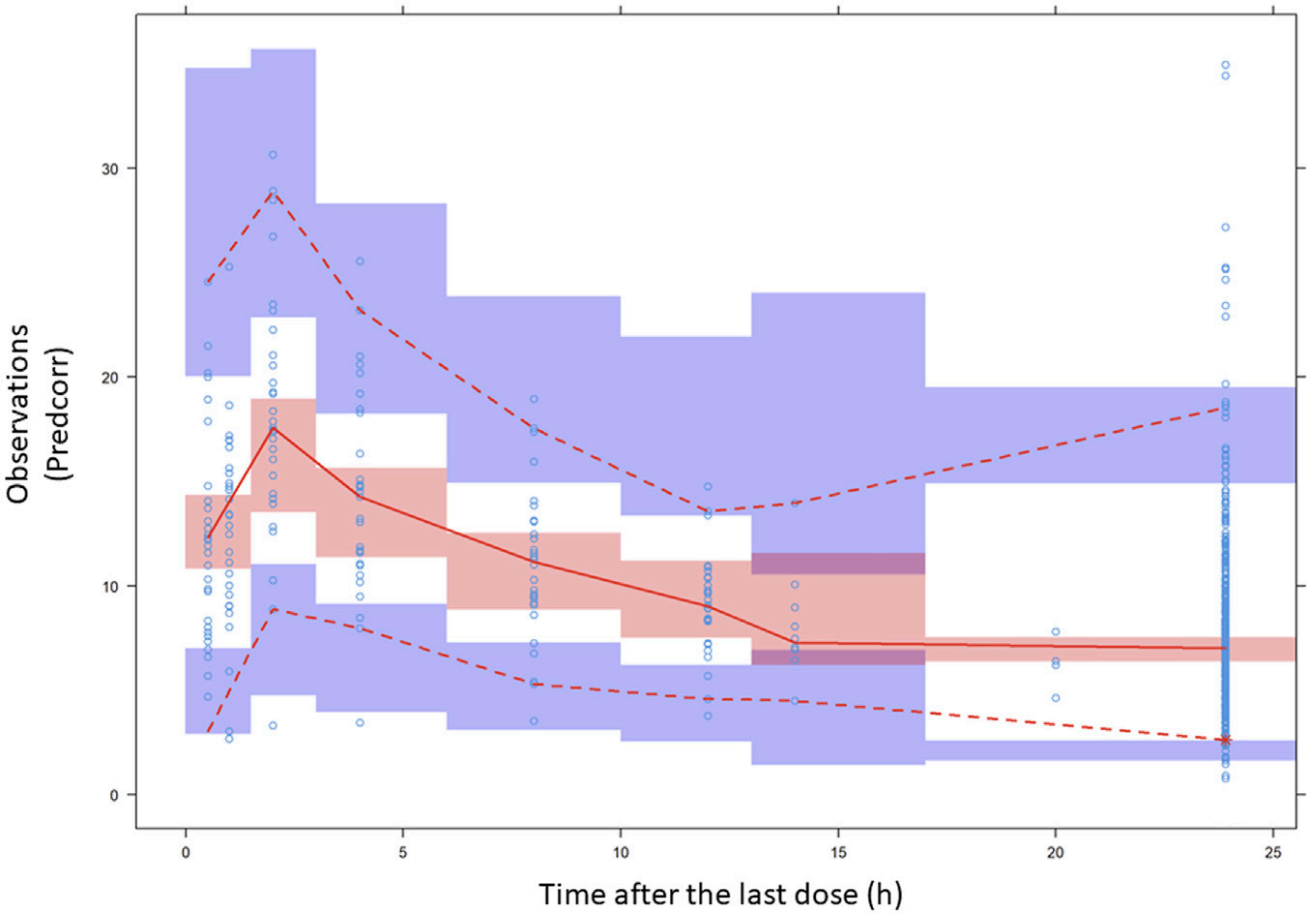
Prediction-corrected VPC results. Prediction-corrected visual predictive check of tacrolimus blood concentration (expressed as ng/mL) vs. time (time after the last dose given in h) profiles. Open circles represent the prediction-corrected observed concentration. In general, median (solid red line), 95th, and fifth percentiles (dashed red lines) of the observations, as well as the 90% confidence intervals (CI) for the median, 5th, and 95th percentiles of the simulated profiles (covered by the light blue areas) are superimposed in each graph. The fifth, 50th, and 95th percentile lines of the observations fell inside the area of the corresponding 90% CI.

The population parameter values of the final model presented in Table 4 were estimated with acceptable accuracy, given by the relative standard error (RSE), except for the BPV associated with Vc/F (RSE:75.4%). This finding was in agreement with the 95% confidence intervals of bootstrap results (Table 4). The stability of the model was confirmed as the bootstrap median values deviated by less than 8% from the final population estimates for all the parameters. The final population parameter estimates were always within the 95% confidence interval obtained by the non-parametric bootstrap. Acceptable shrinkage values were obtained for eta values associated with CL/F (7.45%) and epsilon values (10.5%), but shrinkage was higher than 30% for etas of Vc/F (54.5%). Residual unexplained variability of the final model was of 28.2%, confirming that the structural part of the model adequately described the data. The condition number was less than 10^n^, where n is the number of model parameters (CN = 39.6. Goodness of fit plots, Supplementary Figure S3), which confirmed the descriptive capability of the model, showing no relevant bias in any case. The plots of observed concentrations versus population predictions or individual predictions showed a random distribution around the identity line. Similarly, the conditional weighted population residuals were within the ±4 range and showed a random distribution around zero. The prediction corrected visual predictive check (Figure 2) suggested that the model adequately predicted the observed data over the 24-h dosing interval.

**TABLE 4:** Tacrolimus population pharmacokinetic parameter estimates and bootstrap results for the final model^a^.

Parameter	Units	Final model parameter estimate *(RSE%)*	Median (95% CI) bootstrap result**
Disposition parameters			
CL/F $=\left(\theta 1\cdot \theta 2-\theta 3\cdot \text{AGE}\cdot \text{agecat}\right)\cdot {\left(BW/70\right)}^{0.75}$			
θ_1_	L/h/70 kg	26.5 (7.8%)	26.4 (22.7–30.7)
θ_2_	—	0.666 (8.1%)	0.663 (0.579–0.759)
θ3	L/h/70 kg/y	0.0562(26.0%)	0.0560 (0.0266–0.0863)
Vc/F	L/70 kg	327(14.0%))	326 (259–479)
Vp/F	L	298 (17.4%)	304 (164–468)
CL_D_/F	L/h/70 kg	51.9 (27.0%)	53.5 (29.9–98.5)
Absorption parameters			
Ka	h^−1^	2.0 FIX	—
Lag-time	h	0.341 (9.4%)	0.346 (0.255–0.413)
Between-patient variability			
ω^2^_CL_	%	32.1(15.1%)	31.5 (26.9–37.2)
ω^2^_Vc_	%	48.2(75.4%)	46.4 (19.2–99.9)
Residual variability	%	28.2(3.8%)	27.7 (24.8–30.1)

^a^ CL/F, apparent blood elimination clearance; CL_D_/F, apparent inter-compartmental clearance between central and peripheral compartments; Vc/F and Vp/F, apparent distribution volumes of the central and peripheral compartments; Ka, first-order absorption rate constant; F, bioavailability; σ2, proportional residual variability expressed as the coefficient of variation; ω2, variance of between patient variabilities associated with the PK parameters, expressed as the coefficient of variation. θ2, change of CL/F values in CYP3A5*1 non-carriers with respect to CL/F values in CYP3A5*1 carriers; BW, bodyweight in kg; AGE, age in years; agecat, categorical variable that equals 0 for patients 60 years old and younger and equals 1 for patients older than 60 years; CL/F, CL_D_/F, and Vc/F were allometrically scaled to 70 kg of bodyweight; allometric exponents were fixed to 0.75 for flow pharmacokinetic parameters and to 1 for central compartment distribution volume as follows, CL/F $=\left(\theta 1\cdot \theta 2-\theta 3\cdot \text{AGE}\cdot \text{agecat}\right)\cdot {\left(BW/70\right)}^{0.75}$ ; CL_D_/F $=\theta 5\cdot {\left(BW/70\right)}^{0.75}$ ; Vc/F $=\theta 4\cdot \left(BW/70\right)$ .

**Estimated from 500 re-samplings with the non-parametric bootstrap method.

### 3.3 Model simulations

**FIGURE 3:**
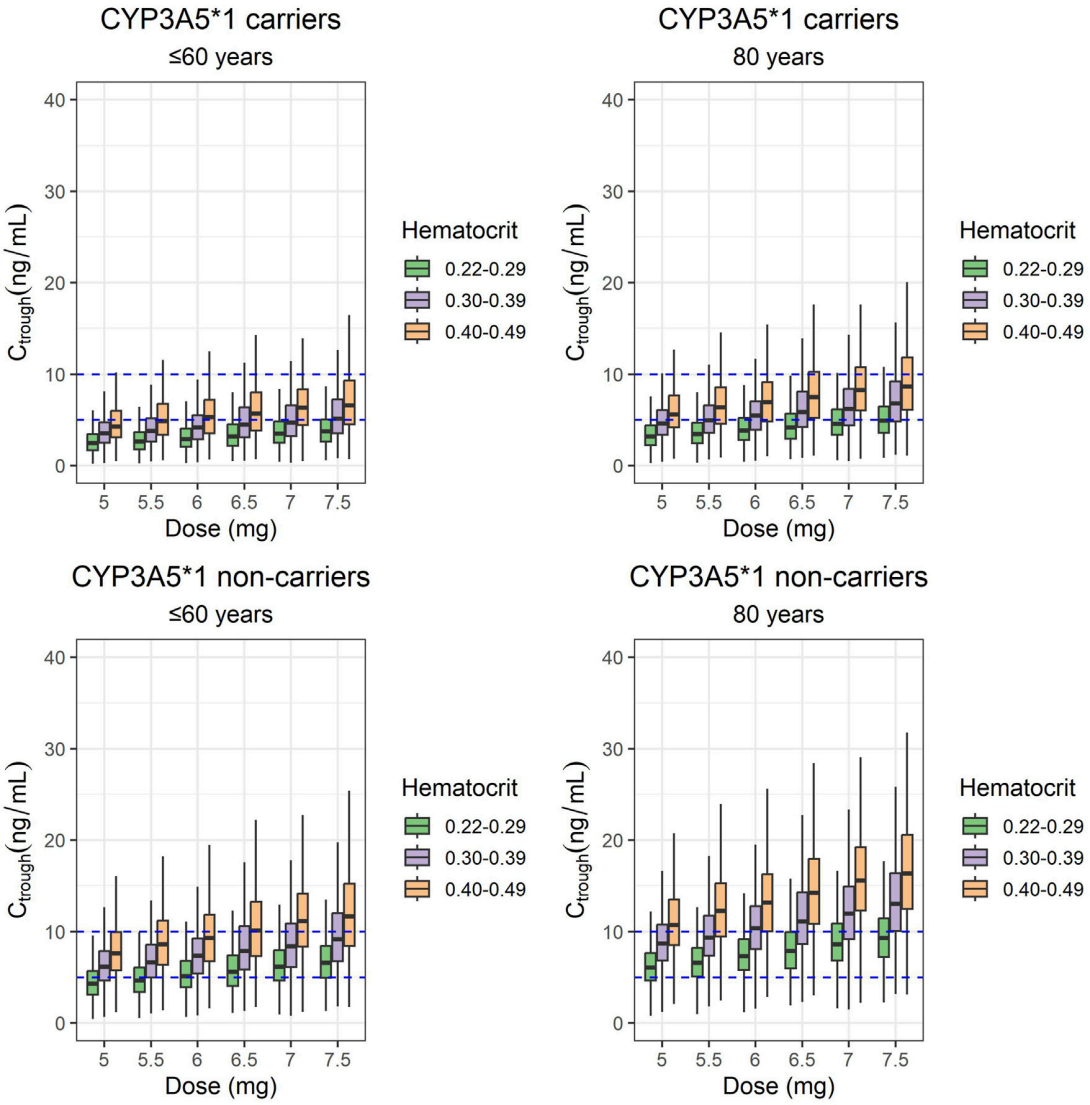
Box plots of C_trough_ simulated values on day 5 from the start of the once-daily doses of 5, 5.5, 6, 6.5, 7, and 7.5 mg of the ER-Tac formulation (corresponding to 0.07, 0.08, 0.09, 0.093, 0.100, and 0.110 mg/kg for a typical patient of 70-kg bodyweight; i.e., the mean value of the target population). Concentrations of patients with hematocrit levels ranging from 0.22 to 0.29, 0.30 to 0.39, and 0.40 to 0.49 and ages lower or equal to 60 years and of 80 years were simulated for each genotype (CYP3A5*1 carriers and CYP3A5*1 non-carriers). Upper panels: CYP3A5*1/carriers. Lower panels: CYP3A5 *1/non carriers. Lower and upper box limits represent the first and the third quartile, respectively. Outliers are not shown. The middle solid line is the median. Dashed lines of 5- and 10-ng/mL indicate the therapeutic range considered in our hospital.

**FIGURE 4:**
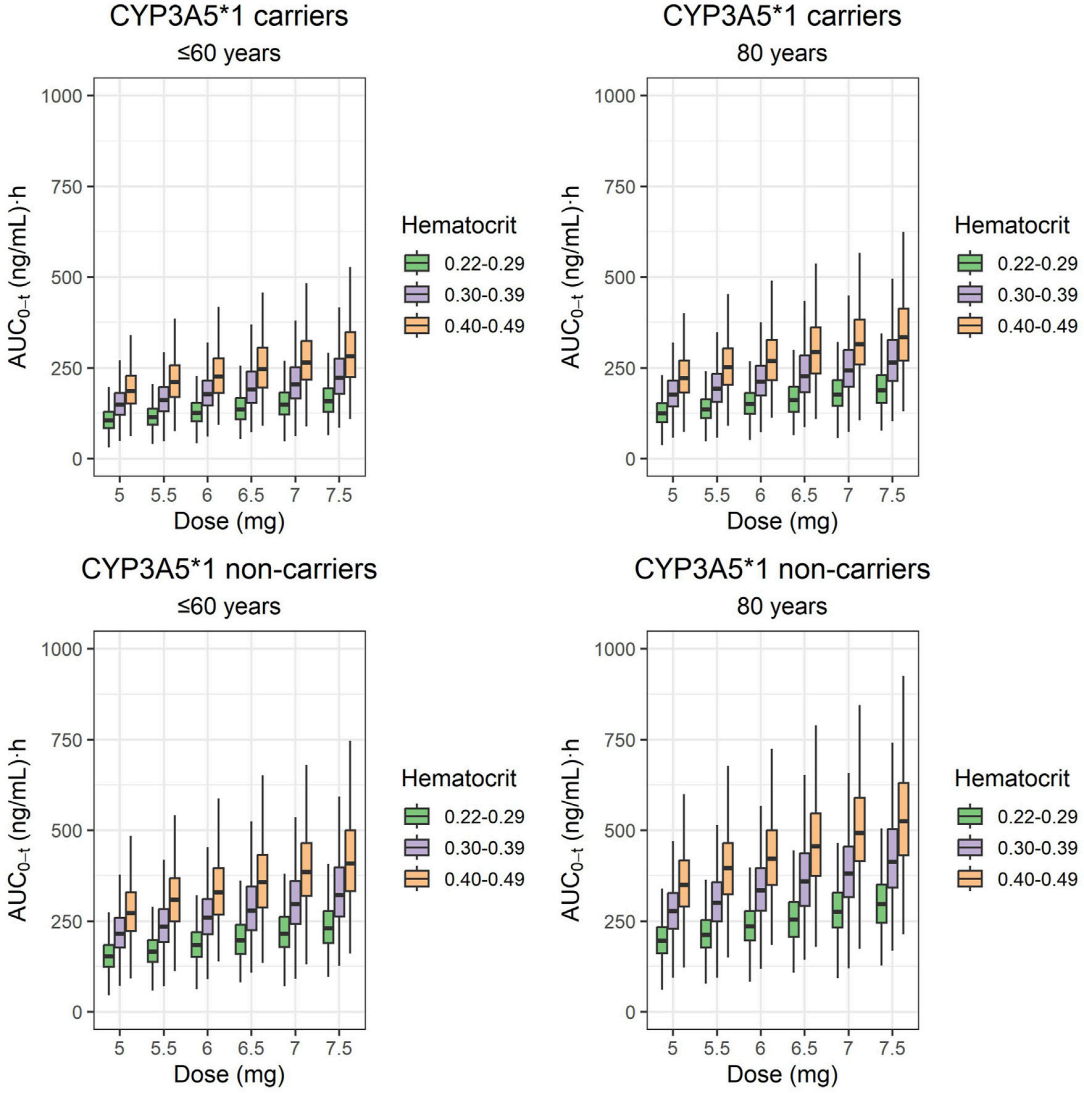
Box plots of AUC_24_ simulated values on day 5 from the start of the once-daily doses of 5, 5.5, 6, 6.5, 7, and 7.5 mg of the ER-Tac formulation (corresponding to 0.07, 0.08, 0.09, 0.093, 0.100, and 0.110 mg/kg for a typical patient of 70-kg body weight; i.e., the mean value of the target population). Concentrations of patients with hematocrit levels ranging from 0.22 to 0.29, 0.30 to 0.39, and 0.40 to 0.49 and ages lower or equal to 60 years and of 80 years were simulated for each genotype (CYP3A5*1 carriers and CYP3A5*1 non-carriers). Upper panels: CYP3A5*1/carriers. Lower panels: CYP3A5*1/non carriers. Lower and upper box limits represent the first and the third quartile, respectively. Outliers are not shown. The solid middle line is the median.

Figures 3, 4 display the boxplots of C_trough_ and AUC_24_ simulated values on day 5 for the once-daily doses of the ER formulation from 0.070 mg/kg to 0.110 mg/kg (equivalent to 5–7.5 mg for a typical patient of 70 kg of body weight). These simulations were performed for patients with hematocrit levels ranging from 0.20 to 0.29, 0.30 to 0.39, and 0.40 to 0.49 and ages 60 years and younger, as well as those aged 80 (which was simulated).

For the same dose, median-simulated C_trough_ and AUC_24_ values were higher for CYP3A5*1 non-carriers than for CYP3A5*1 carriers. In addition, for the same CYP3A5 polymorphism group, the exposure achieved with simulated data (C_trough_ and AUC_24_) was higher for older patients (80 years). Considering the hematocrit values within the normal range (0.40–0.49), CYP3A5*1 carriers aged up to 60 years had a median C_trough_ value within the range of 5–10 ng/mL if they received doses of 6.5–7.5 mg. In contrast, for CYP3A5*1 non-carriers, doses of 5–6 mg were the most adequate among all the simulated doses. CYP3A5*1 carriers aged 80 years would have median C_trough_ values within the therapeutic range at all doses tested, whereas CYP3A5*1 non-carriers would be overexposed in all cases. Simulations of C_trough_ and AUC_24_ corresponding to lower hematocrit values (within the ranges of 0.30–0.39 and 0.20–0.29) resulted in lower total blood exposures than those observed for hematocrit ranges of 0.40–0.49 in all cases.

Simulated median AUC_24_ values were higher than 150 (ng/mL)·h for CYP3A5*1 carriers aged 60 or younger, regardless of the hematocrit values and dose received, except patients treated with 5–6.5 mg and with the lowest hematocrit values. CYP3A5*1 non-carriers aged 60 years or younger tended to show AUC_24_ values over 200 (ng/mL)·h for almost all doses and hematocrit ranges of 0.30–0.39 and 0.40–0.49. The doses of 7–7.5 mg provided median AUC_24_ exposures over 375 (ng/mL)·h in patients with expected normal hematocrit levels (0.40–0.49). CYP3A5*1 non-carriers aged 80 and with normal hematocrit levels had median AUC_24_ values over 250 (ng/mL)·h, regardless of the administered dose.

## 4 Discussion

This study underscores the critical importance of considering the CYP3A5 genotype, age, and the standardization of tacrolimus whole-blood clearance to hematocrit of 45% for guiding tacrolimus dosage during the early post-transplant period. To date, this research is the largest population pharmacokinetic analysis in *de novo* kidney transplant patients treated with the once-daily extended-release formulation of tacrolimus. The pharmacokinetics of tacrolimus given as the ER-Tac formulation was best described by a two-compartmental model with delayed first-order absorption and linear elimination. This finding was in agreement with previous studies (Benkali et al., 2010; Lu et al., 2019; Woillard et al., 2011). However, unlike those studies (Benkali et al., 2010; Saint-Marcoux et al., 2010), the challenge of correctly describing the absorption process could not be achieved, probably due to a more limited sampling design during the absorption phase. Attempts at using gamma distribution to describe the delayed absorption process resulted in unstable, over-parameterized, and poorly conditioned models, and they had to be discarded. This led to differences between Ka and Vc/F values of our model compared to those that used gamma or Erlang distribution to describe the absorption process. Our Ka value (2 h^-1^) was slightly lower than those values obtained with the Erlang distribution (Woillard et al., 2011; Benkali et al., 2010) (5.47 h^-1^ and 3.4 h^-1^, respectively). In contrast, it was higher than the value estimated with the classical lag-time modeling (0.375 h^-1^) (Lu et al., 2019). In addition, a lower value was found for the apparent central compartment distribution volume (327 L/70 kg vs. 486 L (Woillard et al., 2011) and 530 L (Benkali et al., 2010)). In the initial modeling steps, bodyweight was not included in any parameter. However the allometric inclusion of bodyweight, although not statistically significant, contributed to a better prediction of peak and trough concentrations, and it was retained in the final model.

Our results again confirmed the influence of the CYP3A5 genotype on CL/F variability (Andrews et al., 2016). The low prevalence of homozygous CYP3A5*1 carriers only allowed the estimation of the effect of homozygous and heterozygous CYP3A5*1 carriers vs. CYP3A5*1 non-carriers. Specifically, CYP3A5*1 carriers showed a CL/F value 51% higher than that of CYP3A5*1 non-carriers. These results closely resemble those reported for IR-Tac, where the bioavailability (F) of CYP3A5*1 carriers was equal to the 51% of CYP3A5*1 non-carriers, regardless of the effects of age and post-transplant time (Størset et al., 2014a). Conversely, the influence on CL/F, of the genotype cluster variable combining the CYP3A5*3 and CYP3A4*22 SNPs, was not statistically significant. The lack of enough patients with the PM phenotype (n = 3) was probably the reason that led to no differences between CL/F of the poor and intermediate phenotypes among the three of them predefined in the cluster combination (PM, IM, and EM), as occurred with C_trough_ values. By contrast, a different effect on CL/F could be estimated for the EM (n = 47), IM (n = 230), and PM (n = 27) for IR-Tac, in which case, higher sample sizes were available (Andreu et al., 2017). It is worthy of note that the higher CL/F of ER-Tac (26.5 L/h for a CYP3A5*1 carrier) compared to that of IR-Tac could be attributed to a slightly lower bioavailability of ER-Tac.

In the current study, we standardized whole-blood concentrations to a hematocrit of 45% to reduce variability in predicted blood concentrations due to hematocrit differences. Tacrolimus is highly distributed into erythrocytes, with blood/plasma ratios reaching up to 50/1. Consequently, tacrolimus whole-blood concentrations closely reflect concentrations in blood cells. The binding of tacrolimus to erythrocytes and the blood/plasma ratio concentrations increases progressively from the initial post-transplant period onward. As a result of this, and due to its restrictive clearance characteristics, a decline of whole-blood occurs, while therapeutically active unbound concentrations remain relatively stable. Therefore, the standardizing of whole-blood concentrations to a hematocrit of 45% contributes to reducing whole-blood prediction variability caused by the increase of hematocrit during the post-transplant period of the kidney. This standardization can allow a reduction in the risk of inadequate titration during therapeutic drug monitoring. This approach aligns with previous findings (Størset et al., 2014a; Størset et al., 2014b) and enhances the reliability of tacrolimus dosing strategies in the clinical setting. Thus, the standardization led to apparent blood clearance values (CL/F = 26.5 L for CYP3A5*1 carriers) that were lower than those reported for ER-Tac, even in stable kidney transplant patients (42.4 L/h, Woillard et al., 2011; 40.85 L/h; Benkali et al., 2010) and for CYP3A5*1 carriers who had median hematocrit values of 38.5% and did not standardize whole-blood concentrations to a hematocrit of 45%. In contrast, our CL/F values were closer to those found for IR-Tac when the standardization approach was applied. Values of 18.39 L/h for a CYP3A5*1 carrier patient with a fat-free mass of 50 kg (close to the median value in our population) (Størset et al., 2014a) or 20.5 L/h for an extensive metabolizer phenotype of the cluster combination (Andreu et al., 2017) were reported.

As previously pointed out (Andreu et al., 2017), we identified age as a predictor of variability in CL/F. Our current results suggest that significant changes in CL/F take place from the age of 60 onward. This finding is consistent with the fact that the major route of elimination for tacrolimus is by hepatic metabolism. The range of age variation in our study was larger than in some of the previous studies, which likely allowed us to identify the influence of age on CL/F.

Simulations from the final model suggested that considering hematocrit levels of 0.40–0.49, CYP3A5*1 carriers aged 60 or younger showed a tendency to receive a dose too small on the basis of a therapeutic range of 5–10 ng/mL C_trough_ concentrations, when given 5–6 mg (on the basis of 70 kg body weight). CYP3A5*1 carriers aged 80 years had median C_trough_ values within the range (5–10 ng/mL), although doses lower than 5.5 mg tend to cause under-dosing and doses higher than 7 tend to cause overdosing. Conversely, CYP3A5*1 non-carriers aged 60 or younger treated with 5–5.5 mg were correctly exposed but tended to be overdosed with higher doses. CYP3A5*1 non-carriers aged 80 years tended to be overdosed, regardless of the dose given. The simulations also provided information on the therapeutic ranges to be considered for hematocrit values lower than those assumed to be normal (0.30–0.39, 0.20–0.29), to optimize the dose during therapeutic monitoring, in these patient groups.

According to the consensus report (Brunet M et al., 2019), a minimal AUC_0–12h_ threshold of 150 ng·h/mL was proposed for the twice-daily formulation in adult kidney transplantations. In our previous study (Mohammed Ali et al., 2023), AUC_24_ values of 252 and 191 (ng/mL)·h were found after administration of LCP-Tac at doses of 2–12 mg and 0.5–8 mg in stable CYP3A5*1 carriers and CYP3A5*1 non-carriers, respectively. Simulations of our current study showed that doses required in “*de novo*” CYP3A5*1 carriers to achieve median AUC_24_ values of approximately 250 ng·h/mL were approximately 6.5–7.0 mg for patients aged 60 or younger and with hematocrit values expected as normal (0.40–0.49). For patients aged 80 with normal hematocrit values (0.40–0.49), lower doses were required (around 5.5–6 mg). For CYP3A5*1 non-carriers, doses less than 5 mg would be enough to achieve AUC_24_ values at approximately 250 (ng/mL)·h in patients aged 60 or younger, and even much less doses would be enough for patients aged exactly 80, with normal hematocrit levels (0.40–0.49) in all the cases.

The limitations to our study stem from several factors. First, the lack of full pharmacokinetic profiles for all patients included in the study. Second, the limited number of sampling during the absorption phase hindered our ability to characterize the delayed absorption in a more physiologically relevant manner, such as through the use of transit compartment models. The low prevalence of the homozygous *1*1 CYP3A5 variant in the Caucasian target population prevented the showing of the distinct influences of the three CYP3A5 variants (*1*1, *1*3, and *3*3) on the CL/F. Furthermore, no homozygous CYP3A4 *22/22 variants were found in our population. Finally, the low frequency and high variability of CYP3A4*/22 carriers in our population reduced the statistical power of our analysis. Consequently, the combined analysis of CYP3A5*/3 and CYP3A4*/22 SNPs was less robust than that focusing solely on the CYP3A5 SNPs.

Thus, in our newly developed model in *de novo* transplant patients treated with ER-Tac, 36% of between-patient variability in CL/F was explained by CYP3A5 genotype, age, and hematocrit. Hematocrit standardization to 45% explained the variability of tacrolimus whole-blood concentrations, and this was of utmost importance to better interpret whole-blood tacrolimus concentrations during therapeutic drug monitoring. Dose requirements of CYP3A5*/1 carriers who were 60 years old and younger were higher than those of CYP3A5*/1 non-carriers older than 60 years. The current population pharmacokinetic model provides preliminary yet useful insights that can contribute to achieving optimal dosing in this specific population/post-transplant period.

## Data Availability

The raw data supporting the conclusions of this article will be made available by the authors, without undue reservation.
