# Supplementary material for: Guiding the starting dose of the once-daily formulation of tacrolimus in “de novo” adult renal transplant patients: a population approach
Source: Front Pharmacol. 2024 Sep 19;15:1456565. doi: 10.3389/fphar.2024.1456565 (PMC11447946; doi:10.3389/fphar.2024.1456565)
Supplement: Supplementary file 1 [file DataSheet1.PDF]

Supplementary Material

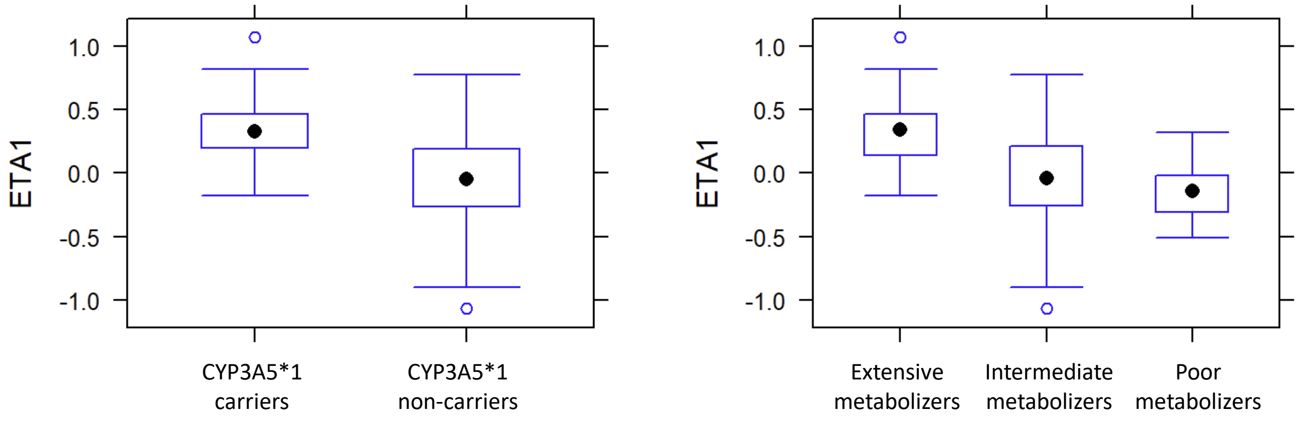

FIGURE S1 (Supplementary material). Boxplots of interindividual random effects associated with CL/F (estimated from the base model) versus a) CYP3A5 genetic variants (Left pannel) b) Cluster phenotypes (Right pannel). Lower and upper box limits represent the first and the third quartile. Data points outside the lower and upper whiskers are outliers. The middle filled circle is the median.

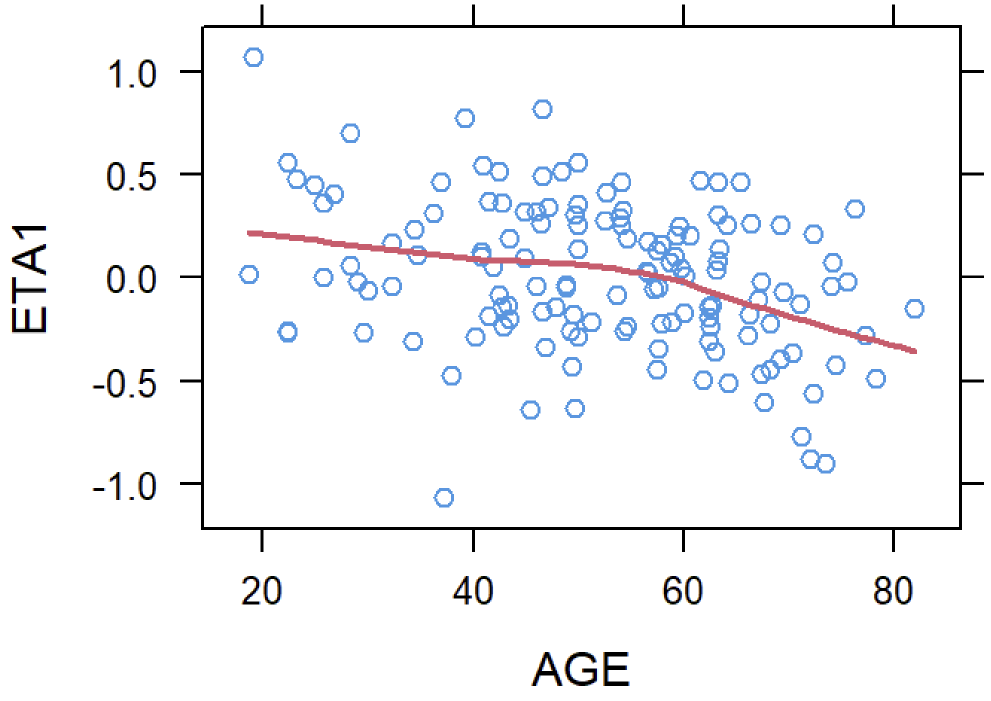

FIGURE S2 (**Supplementary material).** Scatter plot of interindividual random effects associated with CL/F (estimated from the base model) versus age,. Solid red line, Smooth line indicating the general trend of the data.

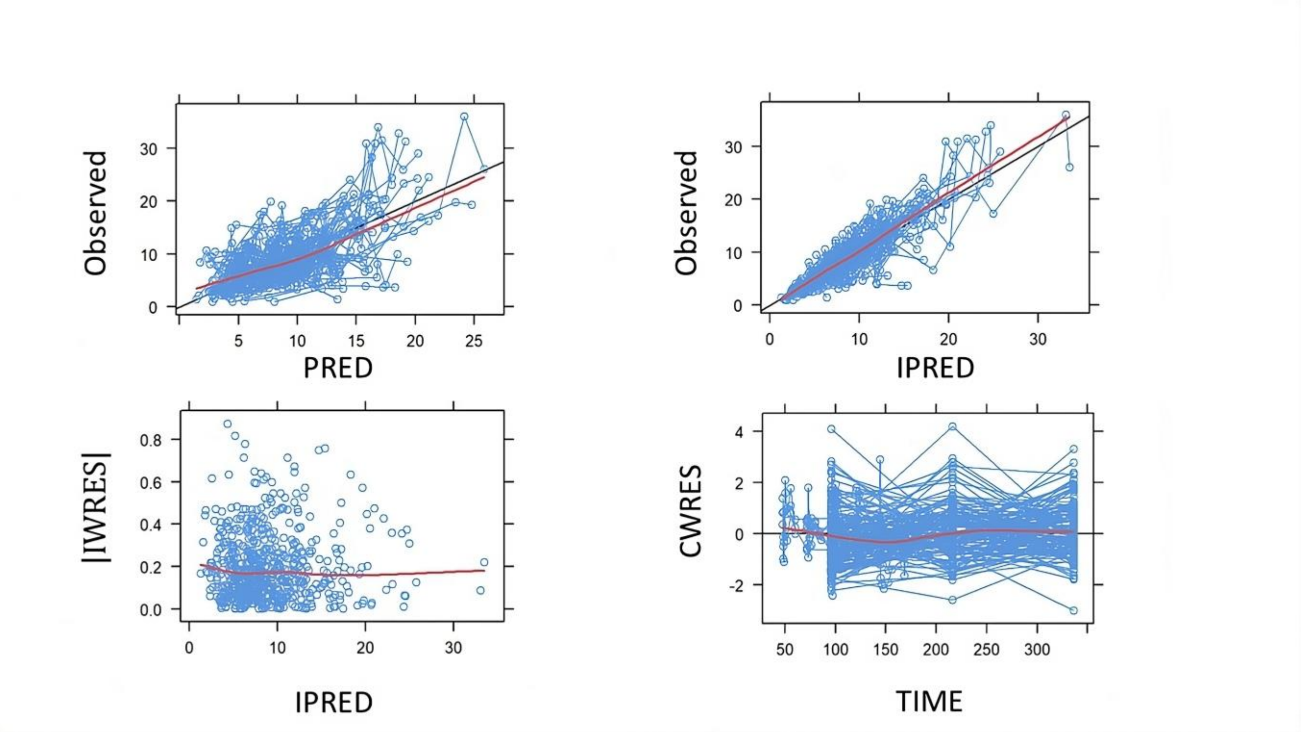

FIGURE S3 (**Supplementary material).** Goodness-of-fit plots for the final model.

Observations: Observed concentrations vs Population predictions (PRED), Left upper pannel; Observed concentrations vs Individual predictions (IPRED), Right upper pannel; Solid black line: Identity line; Solid red line: Smooth line indicating the general data trend of the data. Absolute Individual weighted residuals ($|\boldsymbol{IWRES}|$) vs IPRED, Left lower panel; Conditional population weighted residuals (CWRES) vs time (Right lower panel); Solid black line: it represents the line y=0; Solid red line: Smooth line indicating the general data trend. Concentrations expressed as ng/mL.Time given in hours from the start of the treatment.
